# Self-assembly/disassembly hysteresis of nanoparticles composed of marginally soluble, short elastin-like polypeptides

**DOI:** 10.1186/s12951-018-0342-5

**Published:** 2018-02-17

**Authors:** Markian S. Bahniuk, Abdullah K. Alshememry, Scott V. Elgersma, Larry D. Unsworth

**Affiliations:** 1 Department of Biomedical Engineering, 1098 Research Transition Facility, University of Alberta, 8308-114 Street, Edmonton, AB T6G 2V2 Canada; 2 Faculty of Pharmacy and Pharmaceutical Sciences, 2-35B Medical Sciences Building, University of Alberta, Edmonton, AB T6G 2H1 Canada; 3 Department of Pharmaceutics, College of Pharmacy, King Saud University, Riyadh, Saudi Arabia; 4 Department of Chemical and Materials Engineering, University of Alberta, 12th Floor-Donadeo Innovation Centre for Engineering, 9211-116 Street, Edmonton, AB T6G 1H9 Canada

**Keywords:** Elastin-like polypeptides, Hysteresis, Dynamic light scattering, Inverse temperature transition, Dilution stability

## Abstract

**Background:**

Elastin-like polypeptides (ELPs) are a fascinating biomaterial that has undergone copious development for a variety of therapeutic applications including as a nanoscale drug delivery vehicle. A comprehensive understanding of ELP self-assembly is lacking and this knowledge gap impedes the advancement of ELP-based biomaterials into the clinical realm. The systematic examination of leucine-containing ELPs endeavors to expand existing knowledge about fundamental assembly–disassembly behaviours.

**Results:**

It was observed that these marginally soluble, short ELPs tend to behave consistently with previous observations related to assembly-related ELP phase transitions but deviated in their disassembly. It was found that chain length, concentration and overall sequence hydrophobicity may influence the irreversible formation of sub-micron particles as well as the formation of multi-micron scale, colloidally unstable aggregates. Amino acid composition affected surface charge and packing density of the particles. Particle stability upon dilution was found to vary depending upon chain length and hydrophobicity, with particles composed of longer and/or more hydrophobic ELPs being more resistant to disassembly upon isothermal dilution.

**Conclusions:**

Taken together, these results suggest marginally soluble ELPs may self-assemble but not disassemble as expected and that parameters including particle size, zeta potential and dilution resistance would benefit from widespread systematic evaluations. This information has the potential to reveal novel preparation methods capable of expanding the utility of all existing ELP-based biomaterials.

**Electronic supplementary material:**

The online version of this article (10.1186/s12951-018-0342-5) contains supplementary material, which is available to authorized users.

## Background

Elastin-like polypeptides (ELPs) are a fascinating biomaterial resulting from the fusion of the fields of biology, biochemistry and engineering. Based upon a highly repetitive sequence derived from mammalian elastin, ELPs are a versatile, customizable, stimuli-responsive biopolymer capable of self-assembling into a variety of architectures [1]. The most common ELP is composed of any number of repeats of the pentapeptide sequence valine–proline–glycine–X–glycine (VPGXG) where X can be any amino acid except proline [2]. This sequence can undergo a reversible phase transition in response to environmental stimuli and self-assemble into nano or micron-scale structures. There are a variety of stimuli capable of triggering this assembly including temperature, salt concentration, pH, light and the binding of a ligand, though temperature is the most commonly employed [3, 4]. The sequence and architecture of the ELP construct itself contributes substantially to the exact conditions required to trigger the phase transition, resulting in a flexible and programmable platform technology [5–8]. It is in part due to the relative ease of modification of the ELP family that it is a system under vigorous development for a wide variety of applications. ELPs are being developed for recombinant protein purification, tissue engineering and nanoscale targeted drug delivery [9–11]. There are numerous reviews in the literature detailing the myriad of ways in which ELPs are being engineered for specific applications [11–17].

Despite the widespread development of ELP-based biomaterials, the behaviour of ELPs is still not fully understood. For instance, the mechanism responsible for the reversible phase transition of ELPs is still an area of active investigation. The most recent models suggest that ELPs are intrinsically disordered and capable of momentarily adopting local beta-turn and polyproline structures both below and above their transition temperatures and that sudden decreased backbone solvation may cause ELP aggregation without affecting the structural fluidity of the individual ELP chains [18–24]. Some systematic studies have been carried out examining the effects of guest amino acid, chain length, concentration and pH on the transition temperature (Tt) of ELPs but the scopes of these studies have been limited either by the limited fidelity of early ELP synthetic techniques or by a focus on a narrow range of ELP constructs and particle characteristics [5, 25–33]. While this information may be somewhat useful for designing a construct for a specific temperature or pH trigger using the studied characteristics, these models are still limited in scope and there are other key parameters for which there is no systematic understanding.

Two properties of critical importance to the successful transition of ELP biomaterials to clinical applications like drug delivery include controlling particle size and zeta potential [34]. These parameters are key factors in determining their biological fate and therapeutic efficacy, though no systematic observations have been made on how ELP sequence affects these parameters [34, 35]. Another factor critical to the success of ELPs as a delivery vehicle is their stability upon dilution [36]. Concentration has been shown to affect transition conditions and as clinical administration usually involves diluting these materials within the blood compartment, it is important to understand their disassembly conditions. Other poorly understood aspects of ELP behaviour include whether existing trends in amino acid content, chain length and concentration effects on ELP Tt apply to increasingly hydrophobic constructs, the role of temperature treatment profiles in assembly characteristics, as well as a generalized understanding of ELP behaviour upon cooling.

This study begins to address these unknown parameters while simultaneously expanding upon the existing body of knowledge regarding systematically-studied behaviour of ELPs. The effects of chain length, amino acid chemistry and protein concentration were re-examined using a novel suite of short, highly hydrophobic ELPs developed by our group [7]. ELPs composed of 20, 40, 80 or 160 pentapeptide repeats with leucine in the guest amino acid position, as well as a valine-containing 40-mer control, were studied across a range of concentrations and temperatures using dynamic light scattering (DLS), zeta-potential and transmission electron microscopy (TEM). ELP particle stability upon isothermal dilution was also investigated. The systematic approach undertaken herein allowed for the deconvolution of the effects concentration, guest amino acid chemistry and chain length have on assembly, disassembly and particle characteristics such as size, stability and ELP behaviour as well as advancing the body of evidence supporting clinical therapeutic use of ELPs.

## Methods

### ELP synthesis

ELPs were synthesized according to Bahniuk et al. [7]. Briefly, the initial ELP genes were purchased through Integrated DNA Technologies (Coralville, IA, USA) and were concatemerized together in a pUC-19 cloning vector (Bio Basic, Ontario, Canada) using a modified recursive directional ligation procedure. The restriction enzymes PflMI and BglI (New England Biolabs, Ipswich, MA, USA) were used to create ELP inserts and vectors which were recombined multiple times to form ELP genes of various lengths. Additional digestions, gel purifications and dephosphorylation reactions were employed to ensure a minimum number of *Escherichia coli* (*E. coli*) XL10-Gold (Agilent Technologies, Santa Clara, CA, USA) needed to be screened.

ELP genes were inserted into a pET-25b(+) expression vector containing the N and C-terminal sequences for the ELP genes as well as a tobacco etch virus protease cut site and polyhistidine tag. The expression vector also contained two SfiI (New England Biolabs, Ipswich, MA, USA) restriction enzyme recognition sites with a spacer sequence in order to facilitate efficient vector linearization before ELP gene insertion. As with the concatemerization, additional restriction digests, gel purifications and dephosphorylation reactions ensured the cloning was as efficient as possible. DNA sequencing was carried out at the Molecular Biology Service Unit at the University of Alberta to confirm the ELP genes were correct then the expression plasmids were transformed into OneTouch *E. coli* BL21 (DE3) (Invitrogen, Carlsbad, Ca, USA).

ELP expression was performed by growing the *E. coli* in Terrific Broth (Thermo Fisher Scientific, Waltham, MA, USA) at 37 °C containing 100 µg/mL ampicillin (Thermo Fisher Scientific) and 10 mM l-proline (Sigma-Aldrich, St. Louis, MO, USA) and inducing expression with 2 mM isopropyl β-d-1-thiogalactopyranoside (Thermo Fisher Scientific) once the OD600 of the culture reached 0.8. Upon induction, the cells were left to grow at 37 °C, 225 rpm for 24 h. Cells were pelleted and frozen in liquid nitrogen before being subjected to denaturing metal-affinity chromatography purification. Buffered 8 M urea was used to lyse the cells and fully solubilize any ELP before binding them to nickel beads at 4 °C. After extensive washing the ELPs were eluted using a buffered imidazole step gradient. ELP-containing eluents were subjected to one round of inverse temperature cycling to complete the purification. When necessary, solid NaCl up to 1.5 M and/or temperatures of 37 °C were employed to trigger the phase transition of the ELPs. Exact conditions depended greatly on the construct and its concentration upon expression. No protease digestion was performed on the purified ELPs. Polyacrylamide gel electrophoresis was used to confirm the success of the purifications and sample concentrations were quantified using UV absorbance at 280 nm. The complete amino acid sequence for each ELP construct can be found in Additional file 1: Table S1.

### ELP concentration values

ELP concentrations were normalized by mass in part to control the number of pentameric ELP subunits between samples and also due to technical limitations at both high and low molarities. The lower molarity limit for each ELP sample was dictated by the 0.01 mg/mL minimum sensitivity of the DLS instrument. Given the breadth of ELP lengths studied herein, this meant that the lowest reliably measurable molarity for L20 would be much higher than that of L160. Additionally, attempts to match the highest molarities of shorter constructs using longer ELPs were impeded by the requisite high mass concentrations and difficulties associated with concentrating ELPs to those levels and the resulting instability of the protein solutions, even at low temperatures.

### ELP temperature trend DLS measurements

Thermal behavior of five ELP constructs (V40, L20, L40, L80 and L160) at concentrations of 0.05, 0.1, 0.5 and 1.0 mg/mL was studied using DLS (Malvern Zetasizer Nano ZS, Malvern Instruments Ltd, Malvern UK). Frozen ELP aliquots were thawed and diluted on ice with PBS pH 7.4 to the desired concentration. 100 µL of sample was placed in a 40 µL minimum volume DLS cuvette and kept cold on ice until measurement. ELP samples were equilibrated within the zetasizer at 5 °C before starting measurements. Samples were heated from 5 to 50 °C and subsequently cooled back to 5 °C. Size was measured at 5 °C intervals. A 2 min interval at each measurement temperature was programmed to allow for thermal equilibration of the sample. Two measurements, each with > 10 subruns were recorded at each temperature during heating and cooling. L20 samples at 0.5 and 1.0 mg/mL were sonicated on ice for 30 min prior to the temperature trend measurements to ensure that no particles carried over from the concentrated stock solution or had inadvertently formed during sample preparation. Absorbance, refractive index and viscosity of the PBS and ELP were calculated using Malvern’s zetasizer software 7.03. Protein analysis mode was used for all measurements. Measurement settings were automatically optimized at each temperature interval. Unless otherwise stated, all DLS data represents the peak position of the distribution of particle sizes. All aggregate distributions had polydispersity index (PDI) values < 0.2 unless the samples were actively undergoing a substantial change in particle diameter or the sample had been affected by particle precipitation.

### ELP zeta potential measurements

Zeta potential of five ELP constructs (V40, L20, L40, L80 and L160) at concentrations of 0.05, 0.1, 0.5 and 1.0 mg/mL was determined using a Malvern Zetasizer Nano ZS. All measurements were performed at 5 and 37 °C. Frozen ELP aliquots were thawed in water then placed on ice immediately after thawing. ELPs were diluted with cold PBS pH 7.4 to make two 50 µL aliquots of each concentration. Note that L20 samples at 0.5 and 1.0 mg/mL were sonicated on ice before use as explained previously. All samples were kept on ice until use and ELP solutions, PBS buffer and sample cuvettes were equilibrated at 5 or 37 °C as needed before measurements were taken. A 40 V electrical potential was used to measure all samples. Three measurements of each sample were taken to ensure reproducibility. Each measurement had a minimum of 10 subruns, with a maximum of 60 subruns at 37 °C and 100 subruns at 5 °C. The diffusion barrier technique, details of which are available on the Malvern website, was used for all measurements. 35 µL of ELP was loaded into the bottom of a folded capillary zeta cell (Malvern Instruments Ltd, Malvern UK) already filled with PBS. The sample was then loaded in the instrument and measured immediately to minimize diffusion of the ELP. Absorbance, refractive index, viscosity and dielectric constant of the PBS and ELPs were calculated using Malvern’s zetasizer software 7.03.

### ELP dilution DLS measurements

Nanoparticle size upon dilution of all ELP constructs (V40, L20, L40, L80 and L160) was measured using DLS (Malvern Zetasizer Nano ZS, Malvern Instruments Ltd, Malvern UK). Frozen ELP aliquots were thawed and diluted on ice with PBS pH 7.4 to a starting concentration of 1.0 mg/mL. 100 µL was pipetted into a 40 µL minimum volume DLS cuvette and allowed to equilibrate for 20 min at 37 °C in the zetasizer. Five repeats were collected, each with > 10 subruns used to collect the overall data. This ELP solution was then diluted to 0.5 mg/mL in the DLS cuvette with PBS (isotonic) pH 7.4 kept at 37 °C (isothermal) in a heat block. The sample was then allowed to equilibrate at 37 °C for 10 min before taking 5 measurements. The sample was subsequently diluted to 0.10 mg/mL and then 0.05 mg/mL using this process, taking five measurements at each concentration and allowing 10 min between dilutions. L20 was sonicated on ice for 30 min before measurement in order to ensure no nanoparticles were carried over from the concentrated stock solution or inadvertently formed during sample preparation. Absorbance, refractive index, and viscosity of the PBS and ELP were calculated using Malvern’s Zetasizer Software 7.03.

### Transmission electron microscopy

Transmission electron microscopy (TEM) was carried out at the Cell Imaging Center at the University of Alberta. Samples for TEM were prepared as they were for temperature trend DLS measurements. The samples were heated in a Mastercycler gradient thermocycler (Eppendorf, Hamburg, Germany) under conditions identical to those during the temperature trend. 400 mesh carbon-coated copper grids (Ted Pella, Redding, CA, USA) were subjected to glow discharge in a Pelco easiGlow™ system (Ted Pella, Redding, CA, USA) at 0.4 mBar, 15 mAmp and positive polarity for 45 s. 10 µL samples were immediately placed on charged grids for 3 min incubations on an appropriately warmed heatblock (VWR International, Radnor, Pennsylvania, USA), before two rounds of washing in pre-warmed ultrapure MilliQ water (EMD Millipore, Etobicoke, Ontario, Canada) on the same heated surface. Unstained samples were then directly taken for TEM imaging on a Hitachi H-7650 TEM using a 60 kV accelerating voltage. Images were acquired using a 16 megapixel EMCCD camera (Advanced Microscopy Techniques) and processed using the AMT Image Capture Engine software version 602.576.

### Statistical methods

An unpaired Student’s t-test was used to evaluate the significance of differences between various results. The minimum level of significance was set to α = 0.05.

## Results

**Fig. 1:**
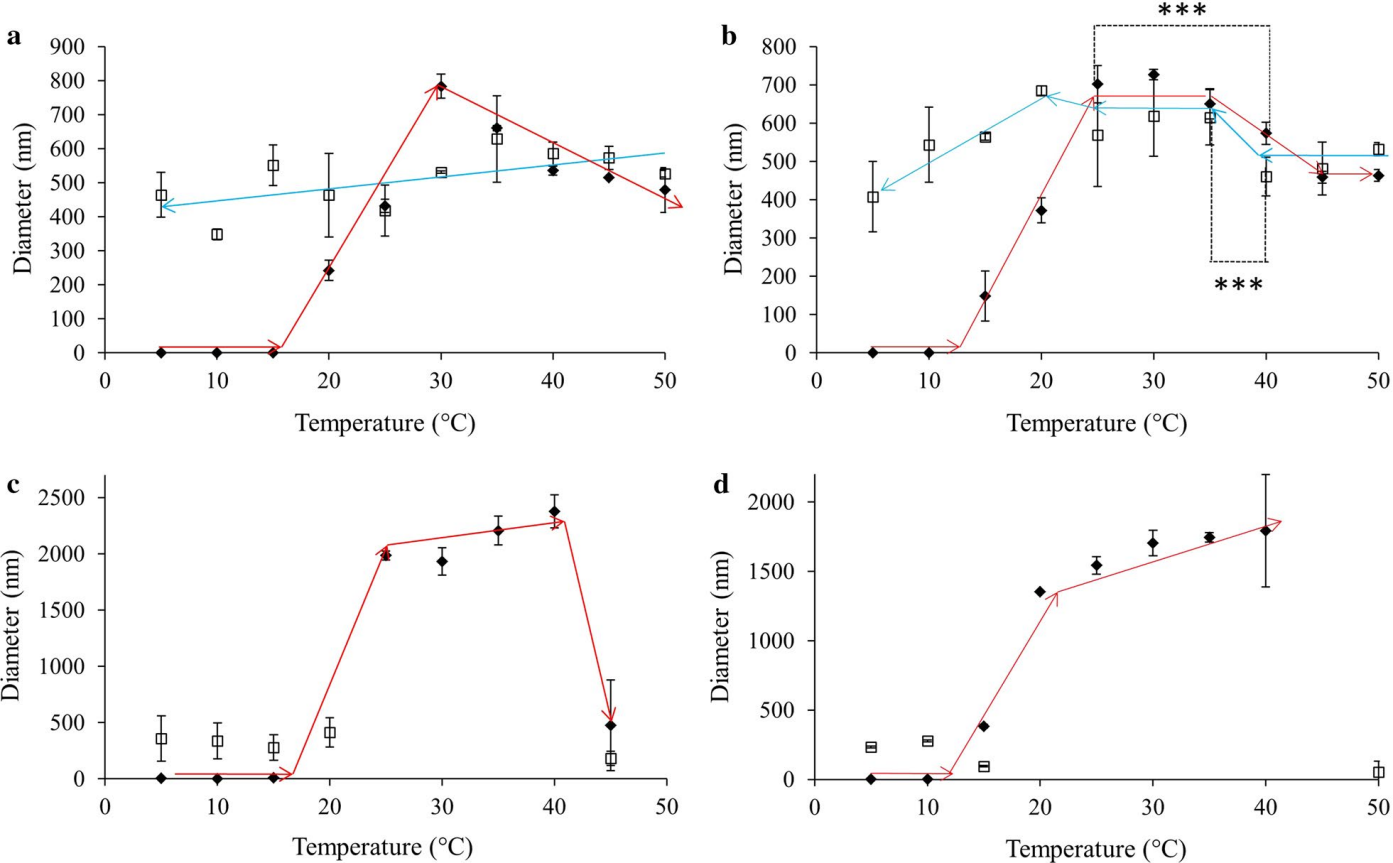
ELP L20 diameters as measured using DLS in 1X PBS pH 7.4, upon heating (filled diamond) and cooling (square). Solution temperature was altered in 5 °C increments with a 2 min equilibration period prior to taking readings. ELP solution concentrations were **a** 0.05, **b** 0.10, **c** 0.50 and **d** 1.0 mg/mL. Missing temperature points were considered unreliable and removed. TEM was used in these instances to analyze particle diameters. Trend lines are presented only to guide the eye; each data point represents the average ± SD, n ≥ 10. *p < 0.05, **p < 0.005 and ***p < 0.0001

The assembly and disassembly of ELP constructs formed from L20 for a variety of conditions are summarized in Fig. 1. Figure 1a summarizes L20 results at a concentration of 0.05 mg/mL exposed to an increase in solution temperature from 5 to 50 °C, which was then decreased back to 5 °C. Upon heating, the diameter suddenly increased from ~ 1 to ~ 240 nm at 20 °C. Aggregate size continued to increase to 780 nm as the temperature was raised to 30 °C, after which the L20 diameter decreased to 478 nm as the temperature increased to 50 °C. Upon cooling, the diameter of the L20 aggregate fluctuated somewhat between 350 and 630 nm but did not return to the original < 10 nm size. The L20 at 0.10 mg/mL (Fig. 1b) showed a similar behaviour to that of 0.05 mg/mL, except the temperature at which the sudden change in size occurred was 15 °C as opposed to 20 °C. The maximal diameter was again observed at 30 °C with a statistically significant decrease in size upon further increase in temperature. Cooling the sample generally reversed the trend seen upon heating from 25 to 50 °C. Further cooling saw the particle size decrease but not return to the pre-heated state.

Increasing the concentration of L20 from 0.10 to 0.50 mg/mL changed the ELP behaviour considerably. Upon heating to 20 °C a drastic increase in particle size was observed (Fig. 1c), with results that were beyond the range of the DLS instrument (> 10 µm). Diameters at higher temperature suggested a particle size in the 2 µm range before decreasing to below 500 nm at 45 °C. Visual examination of the DLS cuvette after reaching 50 °C showed precipitation had occurred during the course of the temperature-dependent measurements. Cooling the sample down to 5 °C did not result in a resolubilization of the precipitated ELPs; supported by the fact that DLS results showed particle diameters did not decrease to < 10 nm. The numerical values for L20 diameters at and above the size shift temperature for the heating as well as the entire cooling may have been affected by the settling of larger particles and the upper sensitivity of the instrument. As such, the data from Panel C should only be interpreted to show that L20 at 0.50 mg/mL had a size shift at 20 °C after which very large aggregates formed and the ELP suspension became unstable. Cooling the sample back to 5 °C did not result in aggregate dissolution. The L20, 1.0 mg/mL system showed a large change in particle size at 15 °C and upon further heating, micron sized aggregates formed that, like the 0.50 mg/mL sample, grew too large to be reliably measured using DLS (Fig. 1d). This sample also displayed instability and precipitated protein was observed after heating to 40 °C and this remained even after cooling back to 5 °C. As such, the data obtained from heating at 45 and 50 °C and the subsequent cooling profile may have been affected by the sample instability.

**Fig. 2:**
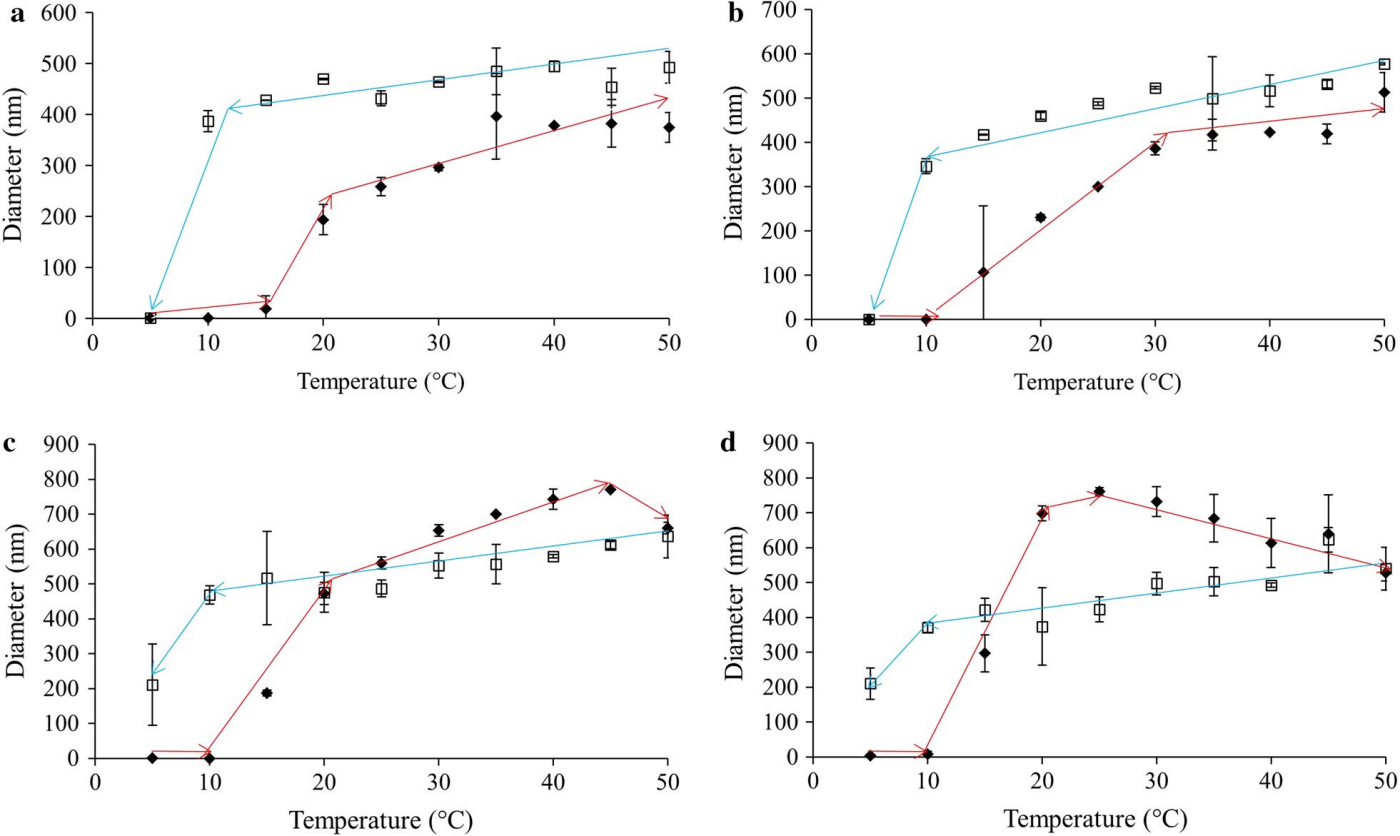
ELP L40 diameters as measured using DLS in 1X PBS pH 7.4, upon heating (filled diamond) and cooling (square). Solution temperature was altered in 5 °C increments with a 2 min equilibration period prior to taking readings. ELP solution concentrations were **a** 0.05, **b** 0.10, **c** 0.50 and **d** 1.0 mg/mL. Trend lines are presented only to guide the eye; each data point represents the average ± SD, n ≥ 10

The behaviour of ELP L40 at various temperatures and concentrations is summarized in Fig. 2. The lowest ELP concentration, 0.05 mg/mL, showed that the particle size drastically changed from approximately 1–200 nm at 20 °C (Fig. 2a). Upon further heating, the diameter increased to about 375 nm. As the sample was subsequently cooled, the diameter of the L40 particles stayed relatively stable until cooled to 5 °C, at which point the ELP aggregates returned to the pre-heated sample sizes. The size of the particles as they were cooled was slightly higher than the diameters observed while the sample was being heated. Similarly, ELPs at 0.10 mg/mL began aggregating at 15 °C and the particle diameter grew steadily with increasing temperature until 30 °C, at which point the size stabilized around 400 nm. At 50 °C the size increased to approximately 500 nm and the diameter decreased slightly but steadily during the cooling treatment. Once the sample reached 5 °C again, the L40 particles appeared to have completely returned to their pre-heated state. L40 ELPs at 0.50 mg/mL showed a change in particle diameter at 15 °C (Fig. 2c). In this case the particle diameter increased steadily, reaching a maximum of ~ 770 nm when the temperature reached 45 °C. Beyond this point and throughout the majority of the cooling treatment the L40 diameter decreased steadily until the sample reached 5 °C when the size decreased sharply, but did not reach a value small enough to indicate a complete return to pre-heated size for the ELPs. At 1.0 mg/mL L40 underwent a transition in particle size at 15 °C which saw the diameter sharply increase to about 750 nm at 25 °C before steadily decreasing in diameter as the temperature increased to 50 °C. Cooling the sample back down continued the trend of a steadily decreasing particle diameter. At 5 °C the size of the L40 decreased sharply from ~ 350 to 200 nm, a behaviour which was also observed for the L40 at 0.50 mg/mL.

**Fig. 3:**
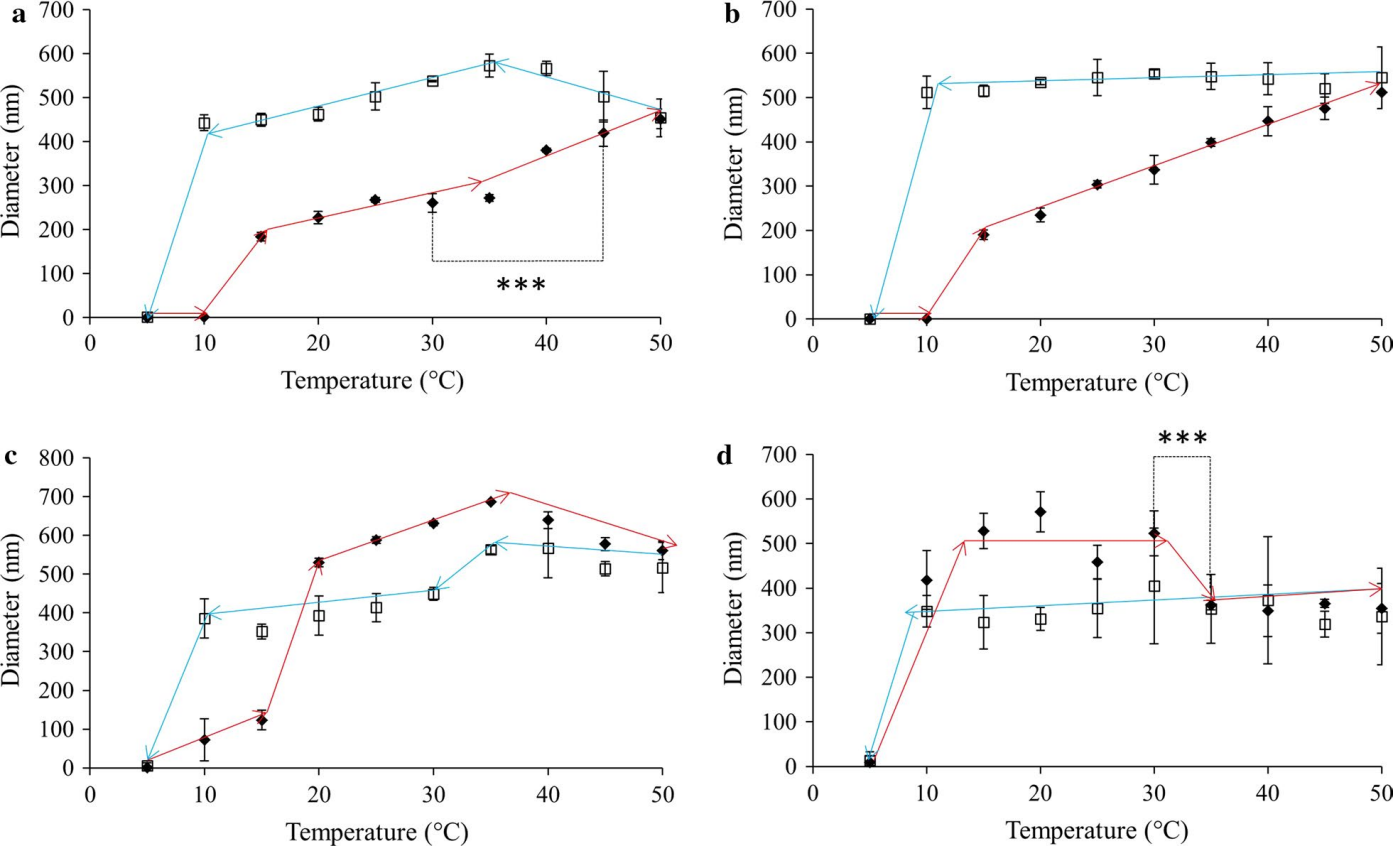
ELP L80 diameters as measured using DLS in 1X PBS pH 7.4, upon heating (filled diamond) and cooling (square). Solution temperature was altered in 5 °C increments with a 2 min equilibration period prior to taking readings. ELP solution concentrations were **a** 0.05, **b** 0.10, **c** 0.50 and **d** 1.0 mg/mL. Trend lines are presented only to guide the eye; each data point represents the average ± SD, n ≥ 10. *p < 0.05, **p < 0.005 and ***p < 0.0001

ELP L80, at a solution concentration 0.05 mg/mL, showed a transition in diameter when heated to 15 °C (Fig. 3a), from 15 to 35 °C the size of the particles remained ~ 240 nm. As the sample was heated further, the ELPs underwent a statistically significant increase in size to about 420 nm. Interestingly, as the sample was then cooled back down to 5 °C, the particle diameter increased to values larger than those seen during the heat treatment, averaging ~ 440 nm until disassembly was observed at 5 °C. Increasing the concentration to 0.10 mg/mL did not result in vastly different behaviour compared to 0.05 mg/mL (Fig. 3b). A similar change in diameter at 15 °C was observed with the particle diameter increased steadily to a maximum of roughly 500 nm at 50 °C. Upon being cooled the particle size remained consistent with the maximal size observed during heating until it returned to its pre-heated size at 5 °C. At 0.50 mg/mL, L80 diameter increased at 10 °C and remained at a diameter of ~ 100 nm until the sample temperature reached 20 °C (Fig. 3c). At this point the diameter sharply increased to more than 500 nm, a range in which the particles remained for the rest of the heat treatment. As this sample was cooled, the particle size fell within the range of 400–500 nm. As seen with the previous L80 samples, this sample also fully returned to its pre-heated size when the temperature reached 5 °C. Finally, at 1.0 mg/mL a transition in diameter occurred at 10 °C (Fig. 3d). Upon reaching this temperature, the particle size underwent a dramatic increase from less than 10 nm to more than 450 nm. The diameter stayed in this range until it was heated to 35 °C, at which point they shrank to about 350 nm-a statistically significant decrease in size. As the samples were cooled, there was minimal variation in the size of the L80 until 5 °C when the particles returned to their pre-heated state.

**Fig. 4:**
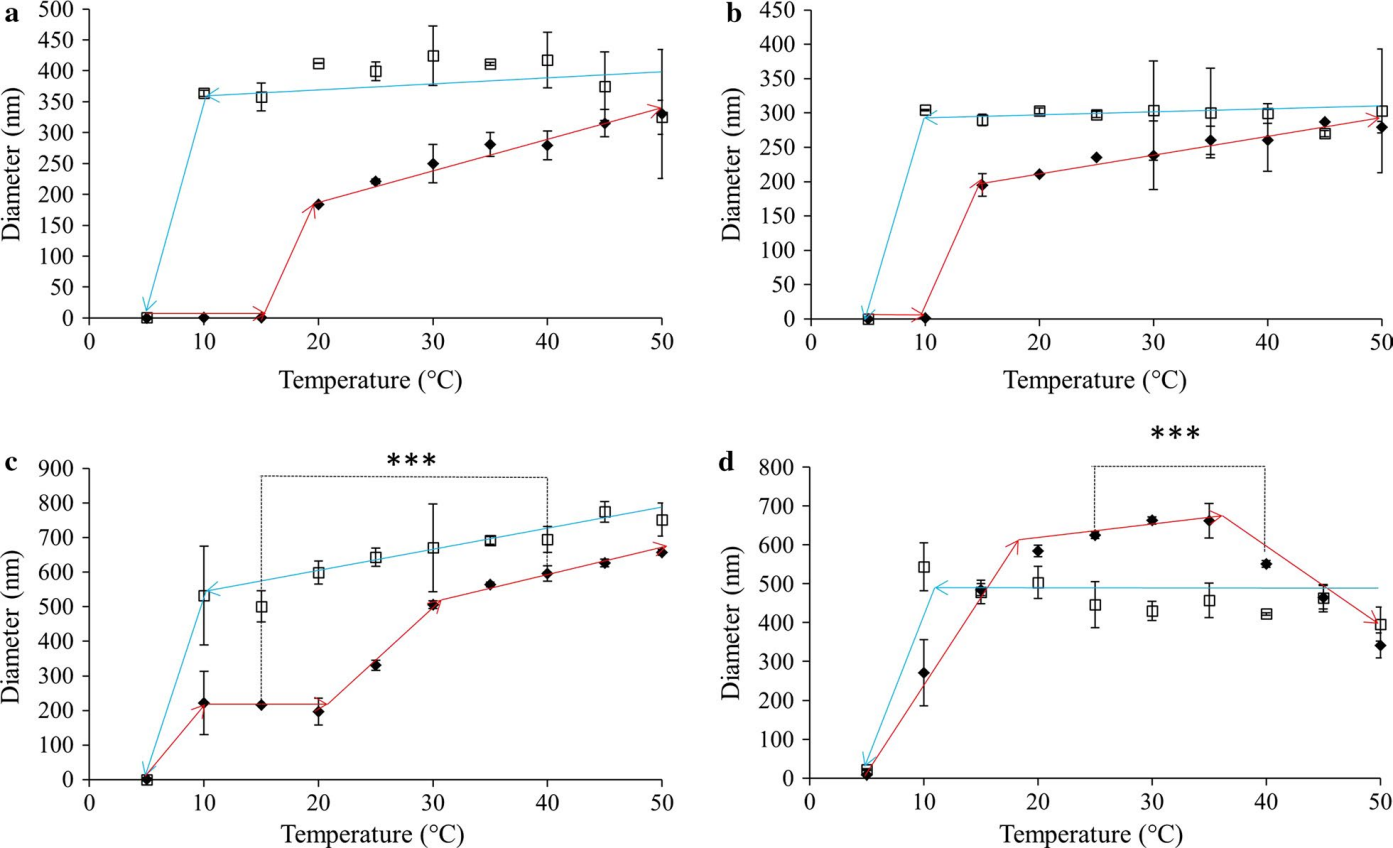
ELP L160 diameters as measured using DLS in 1X PBS pH 7.4, upon heating (filled diamond) and cooling (square). Solution temperature was altered in 5 °C increments with a 2 min equilibration period prior to taking readings. ELP solution concentrations were **a** 0.05, **b** 0.10, **c** 0.50 and **d** 1.0 mg/mL. Trend lines are presented only to guide the eye; each data point represents the average ± SD, n ≥ 10. *p < 0.05, **p < 0.005 and ***p < 0.0001

The results of altering solution temperature and ELP concentration on L160 are shown in Fig. 4. At the lowest concentration, 0.05 mg/mL, the system underwent a transition in diameter at 20 °C, where its diameter increased from ~ 1 to ~ 160 nm (Fig. 4a). As the temperature increased, particle size increased steadily to a maximum of ~ 330 nm. Upon being cooled, the particle diameter did not fluctuate significantly until complete disassembly of the system at 5 °C. When at 0.10 mg/mL the ELPs assembled at 15 °C, with particles at ~ 200 nm that increased to ~ 300 nm upon further heating (Fig. 4b). As the sample was cooled back down to 5 °C, the size of the particles remained constant until complete dissolution was observed at 5 °C. The 0.50 mg/mL solution of ELP L160 transitioned when heated to 10 °C to form particles ~ 200 nm in diameter (Fig. 4c). This size was maintained until heated to 25 °C where the diameter began to increase significantly but steadily, to a maximum of 650 nm at 50 °C. Statistical analysis of the diameters at 15 and 40 °C indicated a significant increase in size upon heating with a p < 0.0001. Upon cooling the particle diameter decreased smoothly to a minimum of about 500 nm before completely returning to their pre-heated state at 5 °C. Finally, a concentration of 1.0 mg/mL showed a size transition at 10 °C (Fig. 4d). When heated to this temperature the particle diameter increased to about 250 nm and continued to grow to a maximum of ~ 660 nm at 35 °C. Further heating of the sample led to a statistically significant (p < 0.0001) decrease in size to ~ 550 nm at 40 °C, a trend which continued to a final diameter of 340 nm at 50 °C. Cooling the L160 solution resulted in a moderate and steady increase in size from 400 nm at 50 °C to ~ 540 nm at 10 °C. At 5 °C the size decreased precipitously, as seen in other cases where the ELP aggregates returned to their pre-heated state.

**Fig. 5:**
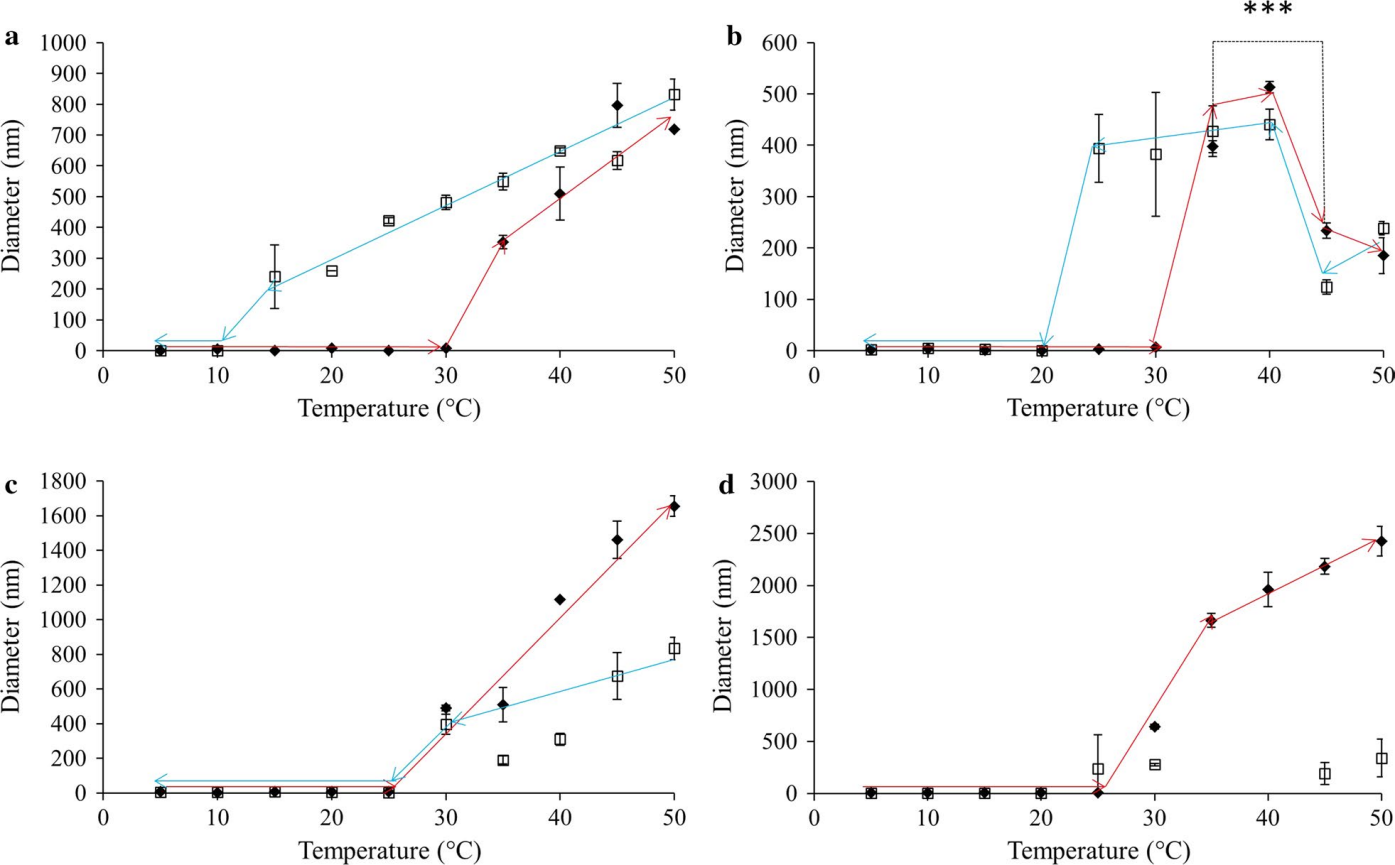
ELP V40 diameters as measured using DLS in 1X PBS pH 7.4, upon heating (filled diamond) and cooling (square). Solution temperature was altered in 5 °C increments with a 2 min equilibration period prior to taking readings. ELP solution concentrations were **a** 0.05, **b** 0.10, **c** 0.50 and **d** 1.0 mg/mL. Missing temperature points were considered unreliable and removed. TEM was used in these instances to analyze particle diameters. Trend lines are presented only to guide the eye; each data point represents the average ± SD, n ≥ 10. *p < 0.05, **p < 0.005 and ***p < 0.0001

ELP V40 assembly as a function of temperature and concentration is shown in Fig. 5. At 0.05 mg/mL this construct underwent a sudden size transition at 35 °C and the size of the particles increased with temperature without reaching a plateau (Fig. 5a). The opposite trend was observed when the sample was cooled, with a complete return to the pre-heated size observed at 10 °C and an apparent hysteresis of 25 °C. Similar to the 0.05 mg/mL results, the 0.10 mg/mL sample underwent a temperature-triggered increase in diameter at 35 °C to ~ 400 nm (Fig. 5b). Once heated to 45 °C a statistically significant reduction in diameter to ~ 200 nm occurred. This behaviour was also observed in reverse when the sample was cooled, with the diameter increasing as the temperature was decreased to 40 °C and a complete return to pre-heated sizes was seen at 20 °C. At a concentration of 0.50 mg/mL V40 began to display unusual behaviour (Fig. 5c). It transitioned at a temperature of 30 °C, but the initial particle size at that temperature was already 500 nm. As the temperature increased the diameter rose into the micron range. Visual observations made on V40 samples after heating to 50 °C showed the presence of some precipitation in the bottom of the DLS cuvette. Once the sample was cooled back to 5 °C, no precipitated material was visible in the bottom of the cuvette. This is consistent with the particle diameter for V40 decreasing to < 10 nm as the sample was cooled. It may be possible however that the diameters recorded during the cooling phase and potentially the dissolution temperature may have been affected by sample instability. This potential instability was further observed when the concentration of V40 was increased to 1.0 mg/mL (Fig. 5d). Like at 0.50 mg/mL, this sample transitioned at 30 °C with an initial particle size of more than 500 nm. The diameter rose to more than 2 μm at 50 °C; again, precipitate was observed after the heating was completed but no insoluble protein was visible upon cooling down to 5 °C. It may be possible that the V40 diameters upon cooling and putative dissolution temperature may have been influenced by sample instability.

**Fig. 6:**
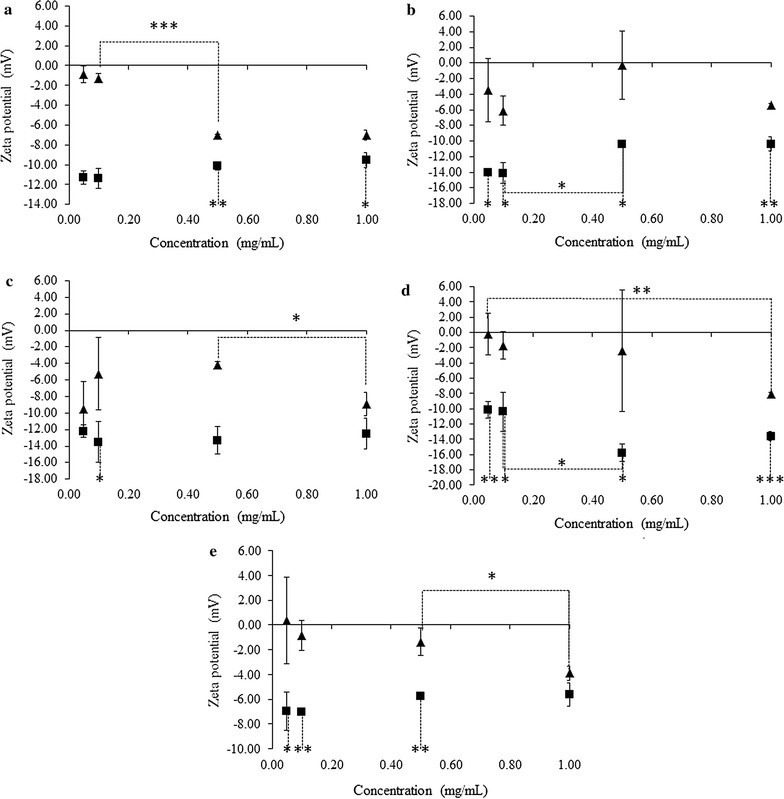
ELP surface charge as measured by zeta potential in 1X PBS pH 7.4, below and above their respective transition temperatures. The constructs are ordered as follows: **a** L20, **b** L40, **c** L80, **d** L160 and **e** V40. Measurements taken at 5 °C are represented by filled triangles while those at 37 °C are represented by filled squares. Reported values represent the mean ± standard deviation with an n = 3. *p < 0.05, **p < 0.005 and ***p < 0.0001

The effect of ELP concentration and sample temperature on zeta potential was also investigated (Fig. 6). The behaviour of ELP L20 can be split into two groups-low concentration samples (0.05 and 0.10 mg/mL) and high concentration samples (0.50 and 1.0 mg/mL). The low concentration samples had a charge of less than − 2 mV when below their assembly temperature and charges of − 11 mV at 37 °C. The high concentration samples had a statistically significantly larger charge below their transition at − 7 mV but at 37 °C had charges in the same range as the low concentration samples at about −10 mV. The change in charge upon heating the high concentration samples to 37 °C was found to be statistically significant for both 0.50 mg/mL (p < 0.005) and 1.0 mg/mL (p < 0.05). Like L20, the low concentration L40 samples (0.05 and 0.10 mg/mL) showed similar charges below and above their assembly temperatures, at about − 4.5 and − 14 mV, respectively (Fig. 6b). Heating these samples from 5 to 37 °C resulted in a statistically significant increase in charge in both cases (p < 0.05). At 0.50 mg/mL and 5 °C the L40 was found to have almost no net charge, while at 37 °C had a charge of − 10 mV, a statistically significant change (p < 0.05). The highest concentration, 1.0 mg/mL, had a stronger charge when disassembled (− 5 mV) and underwent a statistically significant (p < 0.005) increase in charge at 37 °C to a value comparable to that of the 0.50 mg/mL sample. ELP L80 showed similar behaviours between the 0.05 and 1.0 mg/mL samples (Fig. 6c). In both cases the potential at 5 °C was roughly − 9 and − 12 mV at 37 °C. The intermediate concentration samples also had similar characteristics to one another with charges of roughly − 5 and − 14 mV at 5 and 37 °C, respectively. The increase in charge upon heating 0.10 mg/mL samples was found to be statistically significant (p < 0.05). ELP L160 samples at 0.05, 0.10 and 0.50 mg/mL all had similar charges at 5 °C, in the range of 0 to − 2 mV (Fig. 6d). The 1.0 mg/mL sample was significantly more charged at the same temperature, with a charge of − 8 mV. At 37 °C the 0.05 and 0.10 mg/mL samples underwent significant changes in zeta potential values to around − 10 mV (p < 0.005 for 0.05 mg/mL and p < 0.05 for 0.10 mg/mL) while the 0.50 and 1.0 mg/mL samples possessed greater potentials at − 16 and − 14 mV, significant changes from the 5 °C values (p < 0.05 for 0.50 mg/mL and p < 0.0001 for 1.0 mg/mL). ELP V40 showed some similarities to L160 with the 0.05, 0.10 and 0.50 mg/mL samples at 5 °C again falling in the range of 0 to − 2 mV (Fig. 6e). The 1.0 mg/mL sample once again had a significantly larger zeta potential, though only at − 4 mV. At 37 °C regardless of concentration, all V40 samples had similar zeta potentials around − 6.5 mV, changes which were significant compared to the corresponding 5 °C values for all but the 1.0 mg/mL sample (p < 0.05 for 0.05 mg/mL, p < 0.005 for 0.10 mg/mL and p < 0.005 for 0.5 mg/mL).

Generally speaking, when a charge was observed, all ELPs were negative. This is consistent with the average charge of an individual ELP chain being slightly negative. In all cases increasing the temperature from 5 to 37 °C, that is, from below to above Tt for each ELP system resulted in an increase of the magnitude of the zeta potential decrease in the total surface charge of each sample regardless of concentration. This change in zeta potential was found to be statistically significant in many cases with the exceptions of L80 and V40 at 1.0 mg/mL. Concentration likely affected the zeta potential for soluble ELPs, where three of the five constructs tested showed an increasing negative charge. At 37 °C there did not appear to be any discernable relationship between ELP concentration and the zeta potential magnitude.

**Fig. 7:**
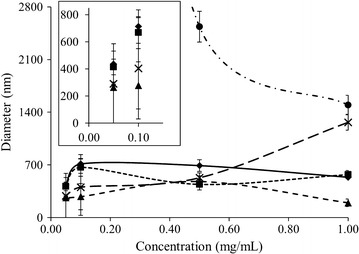
ELP diameters in 1X PBS pH 7.4 at 37 °C as measured using DLS upon dilution from 1.0 mg/mL down to 0.50, 0.10 then 0.05 mg/mL in sequence, using prewarmed buffer. L20 is represented by filled circles with a dash–dot line, L40 by filled triangles and a dashed line, L80 by filled squares and a square dot line, L160 by filled diamonds and a solid line and V40 by × with a long-dashed line. The inset graph is an expanded view of the data points at 0.10 and 0.05 mg/mL. Missing temperature points were larger than the operational range of the instrument. Trend lines are presented only to guide the eye. Values represent the mean ± standard deviation with an n ≥ 10

ELP particle size and stability as a function of dilution was evaluated by diluting 1.0 mg/mL ELP solutions at 37 °C and monitoring particle diameters using DLS. A comparison of the results for each ELP system is given in Fig. 7. ELP L20 displayed some unexpected behaviour when subjected to dilution. At 1.0 mg/mL and 37 °C, L20 had an initial particle diameter of 1.5 µm that increased to 2.5 µm upon dilution to 0.50 mg/mL. Further dilution saw the sizes increase beyond the point where they could be accurately measured with the instrument. Diluting the initial 1.0 mg/mL sample to 0.05 mg/mL did not result in resolubilization as precipitated protein was clearly visible at the end of the dilution testing. ELP L40 had an initial diameter of ~ 200 nm at 1.0 mg/mL, which increased to ~ 480 nm when diluted 2-fold. Particle size decreased to ~ 265 nm for both 0.10 and 0.05 mg/mL. However, the accompanying PDI values increased from < 0.1 to 0.4 and 1.0 upon dilution to 0.10 and 0.05 mg/mL, respectively, which may imply the particle stability was weakened. L80 showed an initial diameter of ~ 570 nm at 1.0 mg/mL that varied moderately over the course of the dilutions to ~ 440, 670 and 415 nm at 0.50, 0.10 and 0.05 mg/mL, respectively. L160 displayed similar behaviour to L80, with a 1.0 mg/mL diameter of ~ 540 nm that, upon dilution, fluctuated to 690, 710 and finally 440 nm as it was diluted to 0.50, 0.10 and 0.05 mg/mL, respectively. Unlike L40, the PDI values for L80 and L160 never exceeded 0.12, a value low enough to imply minimal polydispersity and not large enough to suggest possible perturbation of the ELP particle assemblies. ELP V40 formed particles with diameters of ~ 1.3 µm at 1.0 mg/mL. This value decreased significantly to 540 nm when the sample was diluted to 0.50 mg/mL (p < 0.0001), a trend which continued with values of 400 nm at 0.10 mg/mL and finally 300 nm at 0.05 mg/mL.

Overall the majority of the constructs tended to decrease in diameter as they were diluted from 1.0 to 0.05 mg/mL, the extent of which varied depending on the construct. Only L20 appeared to form larger, irreversibly precipitated particles as the sample became more dilute. Complete particle breakdown was not observed for any system as no diameters below 10 nm were observed; though the PDI for L40 became large enough to suggest some instability in that system upon a 20-fold dilution.

Transmission electron microscopy was carried out as a secondary assessment of ELP particle morphology and diameter. Each construct was examined at 0.10 mg/mL after heating in a manner consistent with previously discussed variable-temperature DLS experiments up to 35 °C. These conditions were chosen as all constructs were stable throughout their heating and cooling at this concentration and the temperature is in the range of biological significance. Additional samples were prepared for L20 and V40 at 1.0 mg/mL after heating to 50 °C in order to visualize the structures formed during precipitation.

**Fig. 8:**
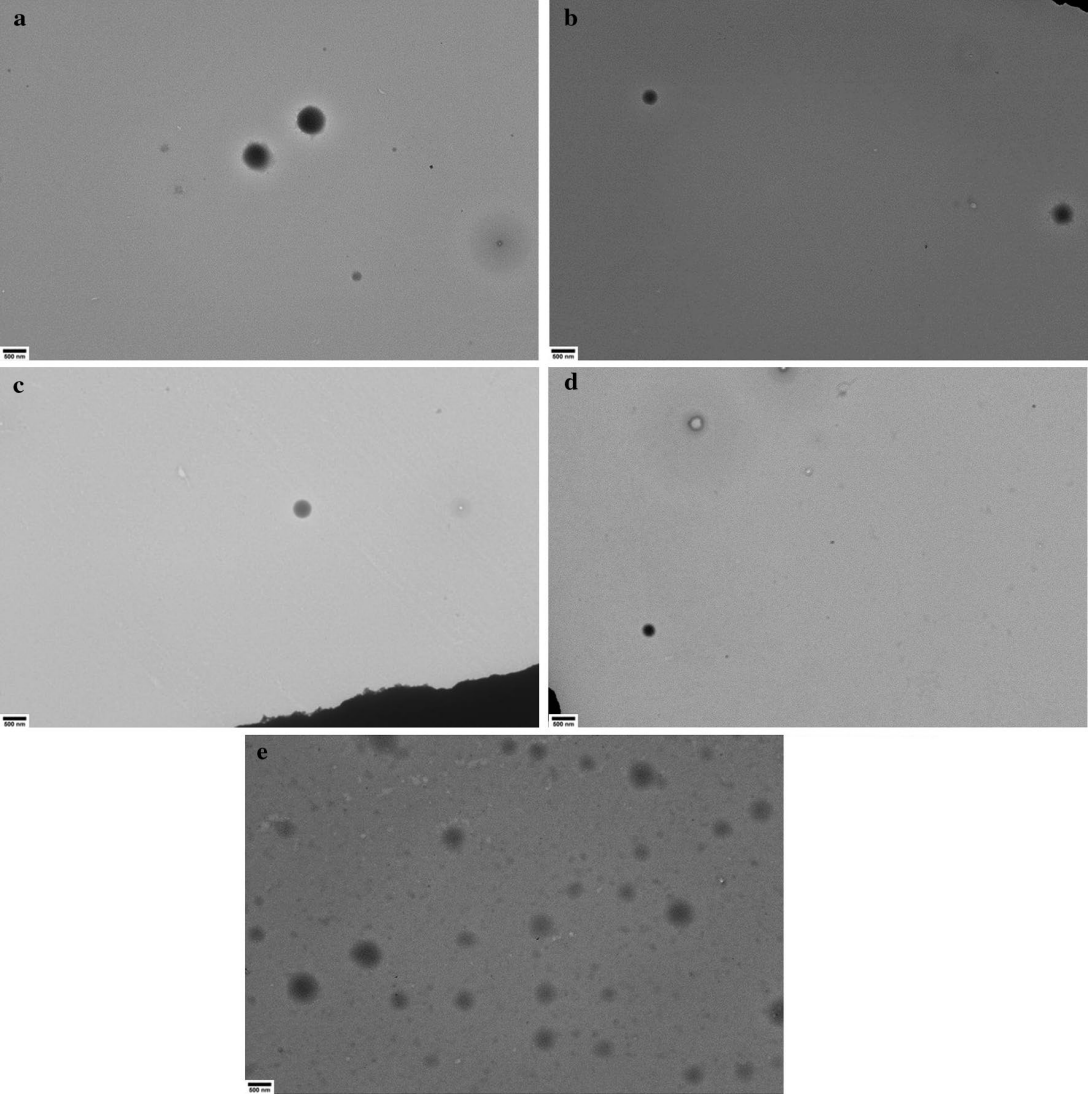
Representative TEM images of **a** L20, **b** L40, **c** L80, **d** L160 and **e** V40 at 0.10 mg/mL after being heated to 35 °C using the identical temperature trend heating profile used for DLS analysis. All images are at ×5000 magnification with a 500 nm scale bar

**Fig. 9:**
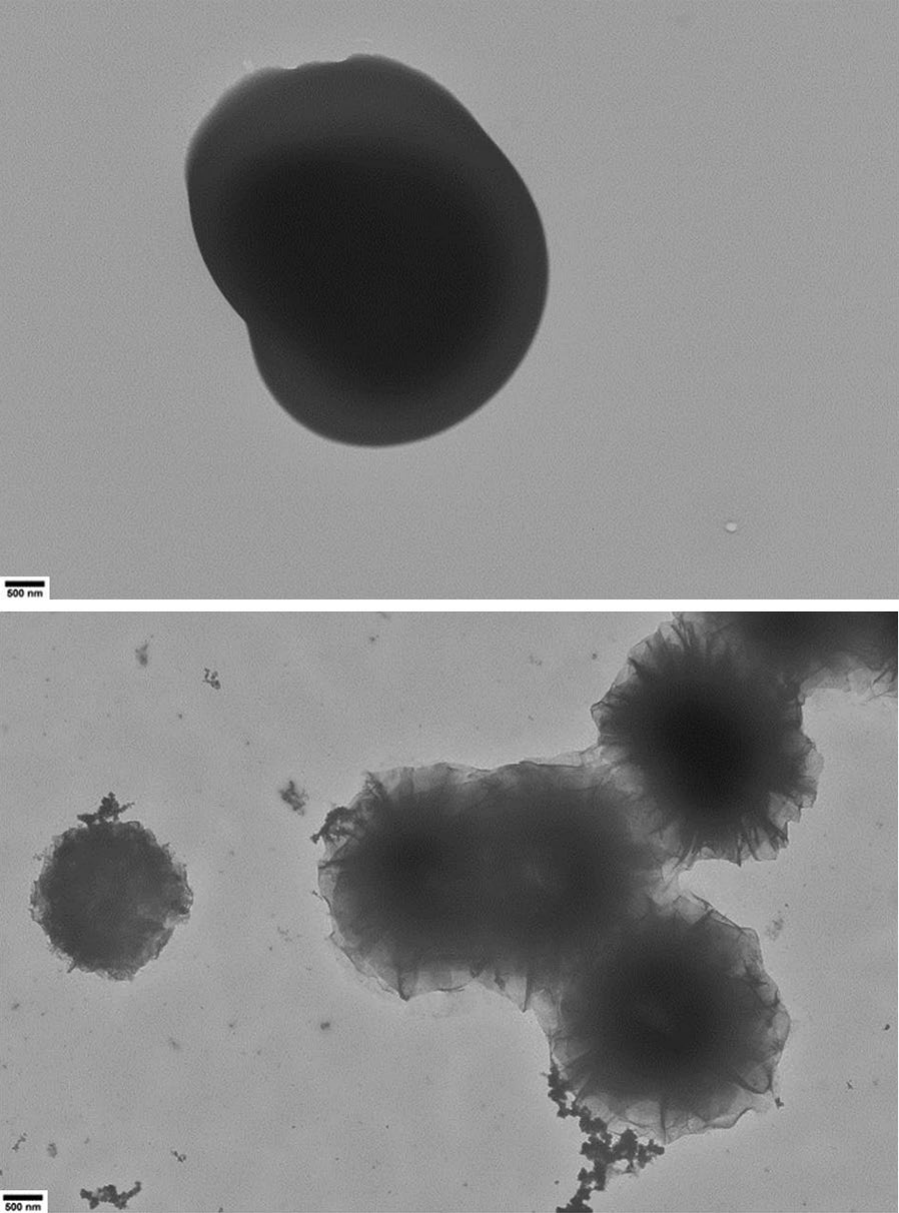
Representative TEM images of L20 (top) and V40 (bottom) at 1.0 mg/mL after being heated to 50 °C using the identical temperature trend heating profile used for DLS analysis. All images are at ×5000 magnification with a 500 nm scale bar

ELP L20 TEM results are shown in Figs. 8 and 9. The lower concentration sample showed particles which were spherical, well dispersed and electron dense. There were many particles visible on each grid and the sizes matched the average measured by DLS, though some heterogeneity in particle diameter was observed. At 1.0 mg/mL there were significantly fewer particles visible on the TEM grids, each with a diameter in the 2–10 μm range. TEM images of ELPs L40, L80 and L160 are all shown in Fig. 8. These three constructs all presented as electron dense, well-dispersed spheres. There were many particles visible for each construct on TEM grids, though more were visible for shorter chain ELPs. The sizes measured by TEM generally matched the results from the DLS at 0.10 mg/mL and 35 °C, with L160 tending to be ~ 20% larger when viewed by TEM than by DLS.

ELP V40 particles at 0.10 mg/mL and 35 °C (Fig. 8) or 1.0 mg/mL and 50 °C (Fig. 9) were similar to the L-series: spherical structures were observed though they did not appear as electron dense as the L-series. At 0.10 mg/mL and 35 °C, the diameters of the V40 particles generally matched those measured with DLS, though the deviations from the mean size appeared larger for the TEM samples. This may be because the standard deviation value reported by the DLS instrument only represents the variance in the mean diameter over multiple measurements, rather than the total width of each measured size distribution. The lower overall charge of V40 particles may have also played a role in that their structures may have been stabilized less by the oppositely charged EM grid than the L-series and, consequently, may have been prone to greater variation in their size once sampled on the EM grid. At 1.0 mg/mL and 50 °C, the V40 structures on the TEM showed the lowest electron density of any sample and the structures resembled spheres that possibly were formed from lamellae of protein. Regardless of formation mechanisms these structures were unlike all other samples.

## Discussion

As explained in the “Methods” section, mass concentration was used to normalize the amount of ELP in each sample due in part to technical limitations. While this allowed for equivalent amounts of ELP pentamers to be present in samples of the same mass concentration, this does not take into account the effect of molarity. As such, considerations of molarity may be used to explain some of the observed behaviours when mass concentration cannot.

With only 20 repeats of the VPGLG amino acid sequence, the L20 ELP is the shortest construct examined with the relatively hydrophobic leucine in the guest amino acid position. Generally speaking, the concentration of L20 did affect the size of the particles formed above the transition temperatures. At a concentration of 0.05 or 0.10 mg/mL, particle size reached a maximum of approximately 650 nm during heating before decreasing to about 500 nm in diameter. At 0.10 mg/mL this decrease in particle diameter while heating as well as the reverse during cooling were found to be statistically significant (p < 0.0001). Increasing the L20 concentration to 0.50 or 1.0 mg/mL resulted in the formation of micron-scale aggregates which were not stable in solution and did not resolubilize upon cooling. The ELP concentration may have some effect on the transition temperature as it varied from 15 to 20 °C depending on the sample conditions, but there was no clear trend with this data set, possibly due to the 5 °C temperature resolution of the DLS program. L20 particle dissolution was not observed at any of the tested concentrations which implies the system exhibits some considerable hysteresis, in the range of 15–20 °C, though it cannot be fully quantified. The diameters of these particles were consistent across DLS and TEM samples. That L20, regardless of concentration, particle size or stability, did not disassemble seriously challenges the fundamental assumption that all ELP phase changes are reversible and that they fully disassemble upon cooling. The irreversible precipitation of L20 was also observed when examining the dilution stability, so the initial concentration-based precipitation is not reversed by sample dilution. This is unlike observations made in this study for V40 under an identical dilution treatment, nor is it consistent with turbidimetric analysis of dilutions performed on particles composed of repeats of elastin exons 20–24 [28]. This property of irreversible precipitation was also seen during routine L20 purifications. Inverse temperature cycling-based purifications of L20 using standard procedures resulted in permanently precipitated samples when ELP concentrations were > 0.40 mg/mL. Additionally, stock solutions of L20 at these high concentrations were found to contain micron-sized aggregates immediately upon thawing (data not shown). These irreversible precipitates could only be successfully resolubilized by minimizing the time they were heated above their Tt during purification and subjecting samples to bath sonication on ice. There is no evidence to suggest that surface charge plays a role in the formation of large L20 aggregates, as zeta-potential values for the system above its transition temperature were all in the range of − 10.5 mV.

Increasing the ELP length from 20 to 40 repeats lead to some differences in the ELP temperature response and assembly behaviour. The concentration of L40 was shown to affect the size of the particles both in the heating and cooling phases. During the heating phase the average particle sizes increased from ~ 350 to 650 nm as the ELP concentration increased while during cooling the average size remained in the 400–450 nm range over the range of the tested concentrations. At no point were micron-sized particles observed. This can also be inferred by comparing the particle size measurements at 50 °C. For L40 the diameters for both heating and cooling are in good agreement, unlike cases when the sample underwent precipitation (i.e. L20 and V40). The Tt was also affected by the ELP concentration. At 0.05 mg/mL the L40 underwent its transition at 20 °C but at 0.10–1.0 mg/mL the ELPs transitioned at 15 °C. Hysteresis of the assembly/disassembly transition temperature was observed for all samples. As the 0.50 and 1.0 mg/mL samples did not fully disassemble upon cooling, a numerical value cannot be determined. For the 0.05 and 0.10 mg/mL samples the values for hysteresis were ~ 15 and 10 °C, respectively. As the more concentrated samples did not disassemble, there may be a concentration-dependence for the dissolution of the L40 system. Particle diameters measured using TEM agreed with DLS results for identical conditions. The zeta potential for L40 when heated above its Tt displayed some concentration dependence, with lower concentration samples having stronger charges. Dilution stability testing did not decisively show particle breakup, though increasing PDI values at 0.05 and 0.10 mg/mL dilutions suggest instability upon further dilution may be possible. This instability upon dilution is not consistent with the behaviour of any of the other ELPs tested herein. While studies of ELP particle stability upon dilution are severely lacking in the literature, Osborne et al. reported that an ELP construct composed of repeats of elastin exons 20–24 demonstrated particle dissolution upon dilution using turbidimetric methods, though the degree of dilution was not reported [28]. ELP L40 behaviour upon dilution appears to fall between the results reported by Osborne and those made here using related L-series constructs.

L80 constructs self-assembled when heated to 10–15 °C to form 200 to 600 nm diameter particles, with both the Tt and particle size dependent upon ELP concentration. Lower concentrations generally resulted in smaller particles, though heating a 1.0 mg/mL solution to greater than 35 °C resulted in a statistically significant (p < 0.0001) decrease in particle size from ~ 500 to ~ 320 nm. Upon cooling, the particle diameters tended to remain steady and upon reaching 5 °C every tested concentration of L80 was shown to fully disassemble. Concentration dependence was observed for the L80 transition temperature during assembly but not disassembly. At 0.05 and 0.10 mg/mL the Tt was 15 °C and at higher concentrations the Tt decreased to 10 °C. Hysteresis was observed under all sample conditions except for 1.0 mg/mL, with a value ranging from 5 to 10 °C. Surface charge and dilution stability data suggest this was a stable construct in general, with no tendency to precipitate or destabilize upon dilution. TEM results confirm the sizes determined using other methods in that L80 presents as a spherical, electron dense particle that was well dispersed and not prone to aggregation.

ELP L160 was the largest tested construct (77 kDa) and in general, the concentration of L160 appears to have some effect on the diameter of the particles formed upon heating. Samples with lower concentrations of ELPs formed smaller diameter particles upon heating, with average diameters in the 250–300 nm range for 0.05 and 0.10 mg/mL samples and diameters in the 500–600 nm range for more concentrated samples. This same trend can be observed for the particle sizes when the samples were being cooled. As with other constructs, some statistically significant (p < 0.0001) decrease in particle diameter was observed for the 1.0 mg/mL sample upon heating above 35 °C. At a concentration of 0.50 mg/mL, L160 formed particles upon heating to 10 °C. The particle diameter remained consistent at ~ 200 nm until the sample temperature reached 25 °C, whereupon the particle diameters steadily increased to a maximum of ~ 625 nm. Cooling the L160 samples back to 5 °C resulted in very little variation in particle diameter until disassembly was observed at 5 °C for all ELP concentrations. Unlike other ELP constructs, each of the L160 samples did show complete disassembly upon cooling in a concentration independent manner. Concentration clearly affected the assembly Tt for this system. Transition temperatures ranged from 10 to 20 °C with more concentrated samples having lower transitions. Hysteresis was observed at 0.05 and 0.10 mg/mL and the value of the hysteresis ranged from 5 to 10 °C. The zeta potential values for this construct above its Tt ranged from − 10 to − 16 mV which suggests these particles should be colloidally stable. This was the only construct to show a greater zeta potential value at 37 °C as the concentration of L160 was increased. Additionally, diluting a sample of L160 particles from 1.0 to 0.05 mg/mL did not result in any meaningful fluctuations of diameter or large standard deviations indicative of decreased particle stability. Electron microscopy was consistent with the observations made with other techniques in that the particles at 35 °C and 0.10 mg/mL roughly matched the DLS observations and resulted in the formation of spherical, electron dense and well-dispersed structures. Previous studies suggested the uniform intensity observed for all L-series ELPs indicated the formation of a micelle-like structure [37].

ELP V40 underwent a phase transition upon heating to 30–35 °C, with higher concentration samples requiring less heating to elicit a response. Once above their transition temperature, V40 formed particles ranging in size from 200 nm to microns in diameter. TEM images suggested that the V40 was not as electron dense as leucine-containing ELPs, which may indicate that guest amino acid hydrophobicity influences packing density. Previous studies have shown that the packing density of assembled ELP above their Tt can be influenced by the phase transition of individual blocks in a block copolymer ELP, solution salt concentration and the distribution of hydrophilic and hydrophobic guest amino acids throughout a multiblock copolymer [27, 38, 39]. The results herein suggest that the role of guest amino acid hydrophobicity in ELP homopolymers may be another avenue by which to engineer the packing density of ELP particles for the needs of specific applications. While there was no clear relationship between ELP concentration and diameter for this system, it was evident that at concentrations of 0.50 and 1.0 mg/mL very large aggregates were formed quickly and precipitation was observed once the samples had been heated to 50 °C. This is consistent with the disagreement between sample diameters at 50 °C during heating and cooling. While this precipitation matches the behaviour for L20, unlike L20 the V40 precipitates resolubilized upon cooling to 5 °C and visible settling was no longer apparent. That this construct readily resolubilized was consistent with its observed behaviour during purification. Inverse temperature cycling procedures were able to precipitate and resolubilize the V40 normally, just like L40, L80 and L160. At 0.05 mg/mL the V40 particle size appeared to continually increase as the sample was heated and the reverse was observed upon cooling. Of all the V40 samples tested, 0.10 mg/mL most resembled the leucine-containing ELPs during its temperature trend. Upon assembly this sample formed structures 400–500 nm in diameter which significantly (p < 0.0001) decreased in size to ~ 175 nm upon heating to 45 °C. As also observed at 0.05 mg/mL, this behaviour was reversed during sample cooling. It appeared that regardless of sample concentration or particle size and solubility, cooling V40 down to as low as 5 °C resulted in complete ELP resolubilization, though large hysteresis values were observed for 0.10 mg/mL and especially 0.05 mg/mL V40. Compared to the leucine-containing ELPs, V40 had lower overall zeta potential values. Above its transition temperature, V40 only had a zeta potential of about − 6.5 mV which did not vary in conjunction with sample concentration. This charge value is low enough that sample precipitation would not be unexpected though precipitation was only observed for the more concentrated samples during the up to 3 h sample testing periods. As with L20 it appears that surface charge is not the only factor that contributes to the development of micron-scale aggregates and subsequent precipitation of ELPs. V40 also demonstrated further unusual behaviour relative to other tested constructs when its stability upon dilution was examined. This construct started off with a diameter of just over 1 μm at 1.0 mg/mL and steadily decreased in size with dilution to a final size of about 300 nm at 0.05 mg/mL, with the first dilution producing a significant decrease in particle diameter (p < 0.0001). Further dilution may result in observations consistent with previously examined ELPs [28]. No other construct demonstrated such a large decrease in diameter. Interestingly, unlike the L20 which precipitated during this procedure, the dilution of V40 appeared to have reversed the growth of the aggregates and prevented their precipitation as no visible settling was observed for this construct. This may be related to the ease with which precipitated V40 can be resolubilized simply by cooling but L20 cannot.

Systematic analysis of ELPs at various concentrations allows for the deconvolution of the effect various parameters have on ELP behaviour in a broader context. Concentration has been shown to influence the formation and disassembly of particles in response to temperature changes. For instance, the degree of hysteresis observed for tested ELPs becomes larger as the ELP concentration decreased (Table 1). The zeta potential of ELPs at 5 °C, that is below their transition temperatures, displayed some tendency to increase in magnitude as the sample concentration increased and this was particularly evident at 1.0 mg/mL. This pattern was not observed for the same ELPs at temperatures above their assembly points. At 37 °C, three of the constructs did not exhibit any variation in charge (L20, L80, and V40), L160 increased with concentration and L40 decreased in zeta potential as concentration increased. Examining the five tested ELP constructs based on solution concentration lead to two distinct groups of behaviours: ELPs which were prone to precipitation (L20 and V40) and ELPs which did not precipitate (L40, L80 and L160). The ELPs which did precipitate demonstrated a previously described transition temperature shift in association with concentration, where a higher concentration resulted in a lower Tt. This group of ELPs also showed a concentration-dependent precipitation where at ELP concentrations below 0.50 mg/mL particles of greater than 1 μm in diameter did not form, nor was precipitation observed either visually or suggested through the DLS results. TEM results were used to confirm the formation of micron-scale aggregates for samples at 1.0 mg/mL and submicron particles at 0.10 mg/mL when the samples were heated above their Tt. Regardless of sample preparation, all particles were roughly spherical. L20 was electron dense and well dispersed, while V40 appeared less dense. Concentration was not observed to independently influence either the tendency or temperature at which L20 and V40 disassembled nor did there appear to be a concentration-dependent particle size at concentrations where precipitation did not occur. The subset of ELP constructs that did not precipitate (L40, L80 and L160) had a variety of responses dependent upon concentration. As observed previously, as the sample concentration increased, the Tt of this subset of ELPs decreased [5, 30, 32]. The disassembly of these constructs did not appear to vary based on sample concentration and was observed at 5 °C for all samples. Generally speaking, more concentrated samples tended to form larger diameter constructs and this relationship was more pronounced while the samples were heated rather than when they were being cooled. Concentrated ELP solutions also tended to show size profiles upon heating with more dynamic behaviour: regions where the size plateaued or increased at varying rates. For L80 and L160 this even resulted in statistically significant decreases in the particle diameters at concentrations of 1.0 mg/mL. TEM confirmed the DLS measurements at 0.10 mg/mL and 35 °C and spherical, well dispersed and electron dense particles were observed for all of the non-precipitating constructs.

**Table 1:** Summaries of ELP assembly and disassembly behaviour upon heating and cooling as observed with DLS in 1X PBS pH 7.4 with heating and cooling in 5 °C increments

Sample (mg/mL)	T_t_ heating (°C)	T_t_ cooling (°C)	Hysteresis (∆ °C)	Zeta potential (5 °C, mV)	Zeta potential (37 °C, mV)	Notes
L20 0.05	20	N/A	20+	− 0.882 ± 0.8	− 11.3 ± 0.6	No disassembly observed
L20 0.10	15	N/A	15+	− 1.27 ± 0.4***^a^	− 11.4 ± 1.0	No disassembly observed
L20 0.50	20	N/A	20+	− 7.04 ± 0.1***^a^	− 10.1 ± 0.4	Not all diameters quantifiable
L20 1.0	15	N/A	15+	− 7.04 ± 0.5	− 9.52 ± 0.8	Not all diameters quantifiable
L40 0.05	20	5	15	− 3.47 ± 4.0	− 14.0 ± 0.5	
L40 0.10	15	5	10	− 6.10 ± 1.9	− 14.1 ± 1.4*^b^	
L40 0.50	15	N/A	15+	− 0.233 ± 4.4	− 10.4 ± 0.2*^b^	No disassembly observed
L40 1.0	15	N/A	15+	− 5.38 ± 0.2	− 10.4 ± 0.9	No disassembly observed
L80 0.05	15	5	10	− 9.53 ± 3.4	− 12.2 ± 0.8	
L80 0.10	15	5	10	− 5.25 ± 4.4	− 13.5 ± 2.5	
L80 0.50	10	5	5	− 4.22 ± 0.5*^c^	− 13.3 ± 1.6	
L80 1.0	10	5	5	− 8.89 ± 1.4*^c^	− 12.5 ± 1.9	
L160 0.05	15	5	10	− 0.242 ± 2.8**^d^	− 10.2 ± 1.1	
L160 0.10	10	5	5	− 1.71 ± 1.8	− 10.4 ± 2.6*^e^	
L160 0.50	10	5	5	− 2.37 ± 8.0	− 15.8 ± 1.1*^e^	
L160 1.0	10	5	5	− 8.06 ± 0.2**^d^	− 13.6 ± 0.6	
V40 0.05	35	10	25	0.344 ± 3.5	− 6.94 ± 1.6	
V40 0.10	35	20	15	− 0.834 ± 1.2	− 7.00 ± 0.14	
V40 0.50	30	25	5	− 1.37 ± 1.1*^f^	− 5.73 ± 0.3	Not all diameters quantifiable
V40 1.0	30	25	5	− 3.90 ± 0.6*^f^	− 5.61 ± 0.9	Not all diameters quantifiable

Zeta potential values represent the mean ± standard deviation with an n = 3

Asterisks denote statistical significance with * p < 0.05, ** p < 0.005 and *** p < 0.0001

The letters a–f denote the pairs of data compared together in order to evaluate statistical significance

By comparing the responses of L20, L40, L80 and L160 at equivalent mass concentrations, the effect of chain length for hydrophobic, short ELPs was elucidated. Consistent with previous reports in the literature, transition temperatures decreased with longer chain lengths, holding all other parameters constant [5]. One of the most interesting results was that chain length may affect the size and stability of particles formed in solution and that the relationship between chain length and particle size is inverse. Longer ELPs were found to form smaller diameter particles when, given identical mass concentrations, there were equal amounts of ELP pentapeptide sequences between samples. The relationship between ELP chain length and particle diameter and the role of the quantity of ELP pentapeptides compared to the number of ELP molecules has never been explored in the literature previously. Longer ELPs appeared to more readily disassemble upon cooling compared to the shorter constructs, with L20 resolubilization not observed under any of the current tested conditions, limited by the lower mass sensitivity of the DLS instrument. The lack of observable L20 resolubilization herein may be explained by the relatively high molarity of the L20 samples compared to the longer ELP constructs. Some hysteresis was present during sample cooling, with larger discrepancies between assembly and disassembly temperatures for the shorter ELPs. The dilution stability results suggested that longer ELPs tended to retain their size better than shorter ones, though the precipitation of L20 during the dilution test limits the range of lengths that could be successfully tested in this manner. There were no discernable relationships between ELP chain length and surface charge, regardless of sample temperature, nor did there appear to be a correlation between length and ELP behaviour in response to cooling. All construct diameters remained stable until cooled to 5 °C. L20 was found to form larger particles in general and, at high concentrations, micron-scale structures which precipitated out of solution. L40, L80 and L160, composed of identical but longer sequences, consistently formed particles with sub-micron diameters without any solution stability issues over the course of the 3 h measurements. TEM observations were consistent with sizes measured using DLS and support the notion that chain length showed an inverse relationship with particle size for the leucine-containing ELPs.

A comparison of the responses of L40 and V40 can be used to extrapolate generalizations regarding the influence of guest amino acid residues. Clearly the more hydrophilic V40 had a higher Tt than the L40. This is generally in agreement with predictions described in previous literature, though there are some variations due to the different lengths of ELPs tested [31]. V40 was also observed to form significantly larger particles upon heating compared to L40. So much so that the V40 even precipitated at 0.50 and 1.0 mg/mL while the L40 formed sub-micron particles with no observed stability issues during the ~ 3 h temperature trend testing. At concentrations where precipitation was not a concern, L40 tended to present as more consistently-sized particles while being heated. While being cooled, V40 sizes resembled those measured during the corresponding heating size trends while L40 particles had a relatively steady diameter with an abrupt change in size at ~ 5 °C. V40 also appeared to have larger hysteresis values than L40 when precipitation was not observed, with differences as large as 25 °C for V40 but only 15 °C for L40. There was no clear relationship between particle size and guest amino acid residue observed. Zeta potential of assembled particles, that is at 37 °C, did appear to be affected by the guest amino acid with the more hydrophobic L40 displaying a stronger negative charge at all concentrations compared to V40. TEM analysis revealed that the V40 construct was less electron dense that L40 which implies that a greater hydrophobicity may result in tighter packing of ELP chains [27].

The systematic analysis of a family of ELPs with varying guest amino acid hydrophobicity and/or chain length at several equivalent concentrations over a range of temperatures allowed for the deconvolution of the influence these various sample parameters have upon particle assembly behaviour and characteristics. This information can be broadly applied to the design of all future ELP-based materials and also can be used to expand the utility of existing ELP constructs. This novel family of hydrophobic ELPs behaved consistently with previously reported observations regarding the effects of guest amino acid chemistry, chain length and concentration on ELP assembly transition temperatures. The disassembly and hysteresis behaviour of this family of ELPs challenges the generalization that VPGXG-type ELPs disassemble and resolubilize upon cooling. Systematic experimentation revealed that chain length, concentration and guest amino acid hydrophobicity may all influence this irreversible assembly. The surface charge of the systems was shown to be influenced by the solubility state of the ELPs as controlled by temperature and also by the guest amino acid hydrophobicity. More hydrophobic guest resides may result in a greater density of ELP molecules per particle, as suggested by the electron densities seen in TEM. The size of the ELP particles was influenced by sample concentration, with higher concentrations associated with larger particles and, in some cases, micron-scale aggregate formation and precipitation. More concentrated samples also showed more dynamic diameters upon heating, with samples at 1.0 mg/mL showing significant decreases in diameter with heating. Chain length also played a role, with shorter chain lengths resulting in larger particles overall and greater tendencies for precipitation. ELP precipitation was observed for two separate constructs and potentially influenced by both guest amino acid hydrophobicity and chain length. More hydrophobic guest amino acids may allow for greater stability for shorter ELP sequences and may also result in a less reversible particle formation and/or precipitation depending on sample concentration. Hysteresis was observed in all samples and, due to its relationship with the Tt, varied with ELP concentration. Additionally, hysteresis was found to be more pronounced overall for constructs with shorter chain lengths. These short ELPs also were not found to disassemble as reliably as their longer counterparts, with sample concentration also influencing measurable resolubilization. Hysteresis has been sparingly observed in other ELP systems, with constructs composed of elastin exons 20–24 or of crosslinked valine-containing ELP microgels both exhibiting minor hysteresis (< Δ10 °C) behaviour [28, 40]. A pronounced ~ 15 °C hysteresis was observed for a VPAVG-derived ELP at 32.1 mg/mL, with structural studies revealing that the additional methyl group on the third amino acid caused less bound water to be associated with the protein when heated above its transition temperature which stabilized the particles and impeded their redissolution [41–43]. It is possible that a similar mechanism is responsible for the hysteresis observed for some leucine-containing ELPs reported herein, as these constructs also contain an additional methyl group and result in a more hydrophobic sequence overall, though further study would be required to demonstrate this. This mechanism may also be responsible for the irreversible assembly observed herein, as a pronounced 15–20 °C hysteresis alongside low temperature heating transition temperatures may have resulted in disassembly transition temperature below the freezing point of water and the operating temperature of the DLS instrument. Novel dilution stability testing suggested that pre-formed ELP particles composed of longer chain lengths were more stable when subjected to isothermal and isotonic dilution. Guest amino acid chemistry may also affect dilution stability with more hydrophobic residues showing smaller variations in diameter.

The roles of temperature and concentration on ELP behaviour have not been systematically analyzed and the results reported herein, in addition to the design guidelines presented above, suggest that existing ELP constructs may be used to form particles of varying diameters, depending upon solution conditions and temperature treatments. This may be a resourceful and cost-effective way of expanding upon the utility of individual ELP constructs without resorting to further sequence modifications.

## Conclusions

In this study, leucine-containing ELPs composed of 20, 40, 80 or 160 repeats of the VPGLG sequence as well as a 40 repeat valine-containing construct were evaluated for their assembly, disassembly, particle size, charge and dilution stability characteristics. These experiments were performed at four distinct concentrations in order to deconvolute the role of sample concentration. Additional analysis allowed for the elucidation of the roles of guest amino acid chemistry and chain length on ELP behaviours.

Leucine-containing ELPs showed assembly transition temperatures dependent upon sample concentration and chain length consistent with other more hydrophilic constructs studied in the literature. Chain length was found to have an inverse relationship with particle diameter, with L20, the shortest construct, forming micron-scale aggregates and precipitating out of solution at high concentrations. This behaviour was also observed for the valine-containing V40 construct despite it being twice the length of L20. The guest amino acid chemistry was found to potentially influence the zeta potential, with more hydrophobic constructs showing greater charge and electron densities suggesting a corresponding effect on packing density. Temperature-mediated disassembly behaviour was also found to vary with chain length whereby the shortest constructs tended to resist disassembly upon cooling as low as 5 °C. L40 demonstrated that sample concentration also played a role in disassembly resistance, as more concentrated samples did not completely resolubilize, while less concentrated samples did fully disassemble. No resistance to disassembly was observed in the valine-containing construct. Dilution-mediated disassembly testing indicated that pre-formed ELP particles could be subjected to isothermal and isotonic dilution up to 20-fold their original concentration.

## Additional file

**Additional file 1: Table S1.** Complete amino acid sequences for characterized ELP constructs.
