# Supplementary material for: Self-assembly/disassembly hysteresis of nanoparticles composed of marginally soluble, short elastin-like polypeptides
Source: J Nanobiotechnology. 2018 Feb 17;16:15. doi: 10.1186/s12951-018-0342-5 (PMC5816514; doi:10.1186/s12951-018-0342-5)
Supplement: Supplementary file 1 — Additional file 1: Table S1. Complete amino acid sequences for characterized ELP constructs. [file 12951_2018_342_MOESM1_ESM.docx]

Additional Table 1. Complete Amino Acid Sequences for Characterized ELP Constructs

| Construct Name | Complete Amino Acid Sequence | Number of Amino Acids | Molecular Weight (Da) |
| --- | --- | --- | --- |
| L20 | MHHHHHHGGENLYFQGGGGPGVG(VPGLG)_10_VPGVG(VPGLG)_10_VPGWP | 133 | 11835.95 |
| L40 | MHHHHHHGGENLYFQGGGGPGVG[(VPGLG)_10_VPGVG]_3_(VPGLG)_10_VPGWP | 243 | 21125.17 |
| L80 | MHHHHHHGGENLYFQGGGGPGVG[(VPGLG)_10_VPGVG]_7_(VPGLG)_10_VPGWP | 463 | 39703.61 |
| L160 | MHHHHHHGGENLYFQGGGGPGVG[(VPGLG)_10_VPGVG]_15_(VPGLG)_10_VPGWP | 903 | 76860.50 |
| V40 | MHHHHHHGGENLYFQGGGGPGVG(VPGVG)_39_VPGWP | 223 | 18926.15 |

Isotopically averaged molecular weights were calculated from the primary amino acid sequences using the ProtParam program available at http://web.expasy.org/protparam/
